# High-sensitivity C-reactive Protein and Regression of Low-grade Squamous Intraepithelial Lesion: The Role of Low-grade Inflammation in Cervical Carcinogenesis

**DOI:** 10.2188/jea.JE20200142

**Published:** 2021-12-05

**Authors:** Sangjeong Ahn, Gi Jeong Kim, Sung-Im Do, Kyungeun Kim, Hyunjoo Lee, In-Gu Do, Dong-Hoon Kim, Seoung Wan Chae, Seungho Ryu, Jin Hee Sohn

**Affiliations:** 1 Department of Pathology, Kangbuk Samsung Hospital, Sungkyunkwan University School of Medicine, Seoul, Republic of Korea; 2 Department of Occupational and Environmental Medicine, Kangbuk Samsung Hospital, Sungkyunkwan University School of Medicine, Seoul, Republic of Korea; 3 Center for Cohort Studies, Total Healthcare Center, Kangbuk Samsung Hospital, Sungkyunkwan University, School of Medicine, Seoul, Republic of Korea; 4 Department of Clinical Research Design & Evaluation, SAIHST, Sungkyunkwan University, Seoul, Republic of Korea

**Keywords:** chronic inflammation, cervix, regression, low-grade squamous intraepithelial lesions, hs-CRP

## Abstract

**Background:**

Inflammation is emerging as a potential mechanism of cervical carcinogenesis. However, few studies have investigated the association between host inflammatory status and the natural course of cervical precursor lesion. The aim of this study was to assess the probability of LSIL regression, associated with an inflammatory biomarker, high-sensitivity C-reactive protein (hs-CRP).

**Methods:**

In a longitudinal cohort study, female participants were examined annually or biannually using cervical cytology between 2006 and 2015. Incident LSIL cases were included in the analysis, with regression defined as at least one consecutive normal cytologic result. A total of 520 women aged 22–64 years were followed up for LSIL regression. The multivariable-adjusted hazard ratios (HRs) for LSIL regression were estimated using a parametric proportional hazards model.

**Results:**

During 827.5 person-years of follow-up, 486 out of 520 subjects (93.5%) showed LSIL regression. After adjusting several important potential confounders, a higher quartile of hs-CRP levels was significantly associated with a lower rate of regression (for quartile 4 vs quartile 1, inverse HR 1.33; 95% CI, 1.04–1.69; *P* for trend = 0.028).

**Conclusions:**

The low rate of spontaneous regression recorded in women with higher hs-CRP lends support to the role of the perturbated host inflammatory status in cervical carcinogenesis, and suggests that hs-CRP level could help monitor LSIL.

## INTRODUCTION

Cervical infection with high-risk types of human papillomavirus (HPV) is the cause of most cervical cancers and their precursors.^1^ With the advent of HPV vaccinations and cervical cytology screening programs, cervical cancers are now considered preventable diseases.^2^^,^^3^ However, cervical malignancy is still a major public health issue in many developing or underdeveloped countries, and one of the most prevalent female malignancies worldwide.^4^

Low-grade squamous intraepithelial lesion (LSIL) development, a cytologic manifestation of HPV infection, is a distinct event in the natural history of HPV infection and cancer development.^5^ In most women with LSIL cytology, spontaneous regressions occur, but not at the same rate for all women.^6^^–^^14^ Risk factors for cervical precursor progression include smoking, long-term oral contraceptive use, multiparity, and lack of cytology-based screening.^15^^–^^17^ However, the biological behavior of LSIL cannot be completely explained by the conventional risk factors.

Inflammation has emerged as a potential mechanism of cancer development,^18^^–^^20^ including cervical cancer.^21^^,^^22^ Recently, a gene signature for low-grade inflammatory status, which is an intermediate state between tissue homeostasis and classic inflammation,^23^ was found in several cancers, suggesting that inflammatory status plays a role in carcinogenesis.^24^ In cervical cancer, only a few studies have explored the associations between systemic^25^^–^^30^ or cervical (local) inflammation^31^^–^^34^ and the risk of persistent HPV infection and/or cervical precursor progression, exhibiting a positive correlation.^25^^–^^34^ However, the evaluation of low-grade inflammation associated with LSIL regression, which is important in the onset of cervical cancer, is still lacking.

High-sensitivity C-reactive protein (hs-CRP) is one of the most frequently used systemic inflammatory biomarkers. Recent studies have suggested that it has a positive association with cancer.^35^^–^^38^ However, there have been no studies examining how hs-CRP affects cervical lesions. Herein, we investigated the biological behavior of LSIL in a large cohort of participants with incident cytologic LSIL, according to an inflammatory biomarker, hs-CRP.

## METHODS

### Study population

Kangbuk Samsung Health Study is a cohort study of Korean men and women who underwent a comprehensive annual or biennial health examination at Kangbuk Samsung Hospital Total Healthcare Centers in Seoul and Suwon, South Korea.^39^^,^^40^ Over 80% of participants were employees of various companies and local governmental organizations or their spouses since the Industrial Safety and Health Law in South Korea requires annual or biennial health-screening exams, offered free of charge, for all employees. The remaining participants voluntarily purchased screening examinations at the health-screening center.

The present analysis includes a portion of the Kangbuk Samsung Health Study female participants aged 22 or older with cervical cytology examined between the years 2006 and 2015 (*n* = 229,153).

We recruited incident LSIL participants who had one or more completely negative results and subsequent LSIL during the cervical screening test. We excluded participants who had any of the following conditions at baseline: completely negative results of cervical cytology during follow up (*n* = 225,926); prevalent cervical abnormalities before the baseline visit (*n* = 1,563); history of hysterectomy (*n* = 21,225); a history of malignancy in cervix or uterus (*n* = 1,845); or no follow-up cytology (*n* = 3,569). Because some individuals met more than one exclusion criterion, the total number of subjects with incident LSIL at least one follow-up cervical cytology, was 531. Among them, participants with missing hs-CRP (*n* = 11) were excluded. For the final analysis, a total of 520 subjects with incident LSIL were recruited. This study was approved by the Institutional Review Board of Kangbuk Samsung Hospital (KBSMC 2017-11-014), which waived the requirement for informed consent due to the use of de-identified data obtained as part of routine health screening exams.

### Data collection

Baseline and follow-up examinations were conducted at the clinics of Kangbuk Samsung Hospital Health Screening Center in Seoul and Suwon. Data on demographic characteristics, smoking status, alcohol consumption, physical activity, educational level, medical history, and medication use were collected using standardized, self-administered questionnaires, as previously described.^40^^,^^41^ Smoking status was categorized into never, former, or current smoker, and alcohol consumption into none, moderate (<10 g/day), or high intake (≥10 g/day). The weekly frequency of moderate or vigorous-intensity physical activity was also assessed. Height, weight, and sitting blood pressure (BP) were measured by trained nurses. Body mass index (BMI) was calculated as weight in kilograms divided by height in meters squared and was categorized according to Asian-specific criteria.^42^ Hypertension was defined as systolic blood pressure ≥140 mm Hg, diastolic blood pressure ≥90 mm Hg, or current use of antihypertensive medications.

Serum levels of fasting glucose, total cholesterol, and hs-CRP were measured as previously described.^40^^,^^41^ Serum hs-CRP levels were analyzed via nephelometry using a BNII nephelometer (Dade Behring, Deerfield, IL, USA). Insulin resistance was assessed with the homeostatic model assessment of insulin resistance (HOMA-IR) equation: fasting blood insulin (uU/mL) × fasting blood glucose (mmol/L)/22.5. Diabetes was defined as a fasting serum glucose ≥126 mg/dL or current use of anti-diabetic medications.

The cervical specimens were obtained using a DNA PAP Cervical Sampler and specimen transport medium (Qiagen). All specimens at baseline were tested with the HR Hybrid Capture 2 (HC2) assay (Qiagen, Gaithersburg, MD, USA) according to the manufacturer’s protocol. HC2 is a sandwich-capture molecular hybridization assay that uses chemiluminescent detection to provide a semi-quantitative result. Briefly, HPV DNA was denatured, and then the single-stranded HPV DNA was hybridized with a mixture of single-stranded, full-genomic-length RNA probes specific for 13 HR HPV genotypes: 16, 18, 31, 33, 35, 39, 45, 51, 52, 56, 58, 59, and 68. Measurements below the relative-light-unit (RLU) cutoff of 1.0 were scored as negative. Positive and negative controls provided by the manufacturer were included in each run.

Regression in incident LSIL subjects was defined, based on the characteristics of our database that was from an annual or biennial screening exam, and the natural history of LSIL that is mostly determined within 1–2 years. The regression was defined as at least one consecutive negative result in follow-up cervical cytology. Persistence and progression, which were analyzed together as non-regression, were defined as ongoing abnormal cytologic results, including LSIL or above.

### Statistical analysis

The characteristics of the study participants were explored according to hs-CRP quartiles (≤0.2, 0.3–0.3, 0.4–0.6, or ≥0.7 mg/L). The primary endpoint was LSIL regression, defined as negative cytology at the second visit. Each participant was followed from their baseline exam until either LSIL regression or the last health exam conducted before December 31, 2015, whichever came first. The incidence rate was calculated as the number of incident cases divided by the number of person-years of follow-up. Since LSIL regressions would have occurred sometime between the two visits, but the precise time was unknown, a parametric proportional hazards model was used to account for this type of interval censoring (the *stpm* command in Stata).^43^ In these models, the baseline hazard function was parameterized with restricted cubic splines in log time with four degrees of freedom. The proportional hazards assumption was assessed by examining graphs of the estimated log (−log [survival]).

The hazard ratio (HR) and 95% confidence interval (CI) were calculated for incident LSIL regression according to the serum hs-CRP levels. We then derived an inverse HR (1/HR) to label regression with HR that is directed towards the null (<1.00),^44^ which improves understanding of the results and reduces the confusion. Data were adjusted for age, year of a screening exam, smoking status (never, past, current, or unknown), alcohol intake (0, <10, ≥10 g/day, or unknown), marital status, HPV infection, history of diabetes, and education level (high school graduate or less, community college or university graduate, graduate school or higher, or unknown). To determine linear trends of incidence, the number of quartiles was used as a continuous variable and tested for each model. To explore the shape of the dose-response relationship of hs-CRP levels with the LSIL regression, restricted cubic splines with knots were used at the 5th, 27.5th, 50th, 72.5th, and 95th percentiles of the hs-CRP distribution.

Additional subgroup analyses stratified by age (<50 vs ≥50 years), HPV infection (negative vs positive), smoking status (ever or current smoker vs never smoker), alcohol intake (<10 vs ≥10 g/day), BMI (<23 vs ≥23 kg/m^2^), and HOMA-IR (<2.5 vs ≥2.5) were performed. Interactions between hs-CRP quartiles and subgroup characteristics were tested using likelihood ratio tests comparing models with versus without multiplicative interaction terms.

Statistical analyses were carried out using STATA version 15.0 (StataCorp LP, College Station, TX, USA). All *P*-values less than 0.05 were considered statistically significant.

## RESULTS

Table 1 summarizes the baseline characteristics of the 520 participants of incident LSIL included in the present analysis according to quartiles of hs-CRP. The mean age of the study participants was 41.1 (standard deviation, 5.3) years. Women with higher hs-CRP were more likely to be older, obese, and current smokers. Blood pressure, glucose, and HOMA-IR were positively associated with the hs-CRP level.

**Table 1.** Baseline characteristics of study participants by hs-CRP level

Characteristics	Overall	hs-CRP quartiles				*P* for trend

		Q1 (0.1–0.2 mg/L)	Q2 (0.3–0.3 mg/L)	Q3 (0.4–0.6 mg/L)	Q4 (0.7–15.1 mg/L)	
Number	520	244	75	95	106	<0.001
Age, years^a^	41.1 (5.3)	40.3 (5.2)	41.3 (4.7)	42.4 (6.2)	41.4 (5.0)	0.013
BMI, kg/m^2 a^	21.6 (2.7)	20.8 (2.1)	21.7 (2.2)	22.1 (2.5)	23.2 (3.4)	<0.001
Overweight, %^b^	25.8	13.2	24.0	32.6	50.0	<0.001
Current smoker, %	2.9	0.5	3.2	6.4	5.7	0.006
Alcohol intake, %^c^	14.3	17.2	17.9	7.1	11.3	0.050
Vigorous exercise, %^d^	19.0	19.8	19.4	20.7	15.5	0.487
High education, %^e^	66.1	65.9	67.9	57.4	73.3	0.635
Married, %	93.3	94.1	92.5	93.4	92.3	0.659
Menopause, %	8.5	7.4	6.2	12.3	9.3	0.356
Hypertension, %	4.6	3.3	2.7	6.3	7.6	0.060
Diabetes, %	1.9	0.4	4.0	2.1	3.8	0.051
HPV infection, %	87.4	87.0	87.5	84.7	90.7	0.563
Systolic BP, mm Hg^a^	104.3 (12.3)	103.4 (11.8)	101.3 (9.6)	105.1 (12.7)	108.0 (14.1)	0.001
Glucose, mg/dL^a^	92.1 (10.6)	90.5 (7.6)	92.3 (8.7)	92.3 (7.2)	95.2 (17.5)	<0.001
Total cholesterol, mg/dL^a^	186.2 (28.7)	185.6 (27.5)	187.3 (27.9)	184.9 (30.4)	188.1 (30.8)	0.604
HOMA-IR^f^	1.18 (0.79–1.84)	1.13 (0.77–1.66)	1.15 (0.64–1.95)	1.25 (0.84–1.89)	1.46 (0.93–2.13)	0.002

BMI, body mass index; BP, blood pressure; HOMA-IR, homeostasis model assessment of insulin resistance; HPV, human papillomavirus; hs-CRP, high sensitivity C-reactive protein.

Data are presented as ^a^mean (standard deviation), ^f^median (interquartile range), or percentage.

^b^BMI ≥23 kg/m^2^; ^c^≥10 g of ethanol per day; ^d^≥3 time per week; ^e^≥College graduate.

**Figure 1.**
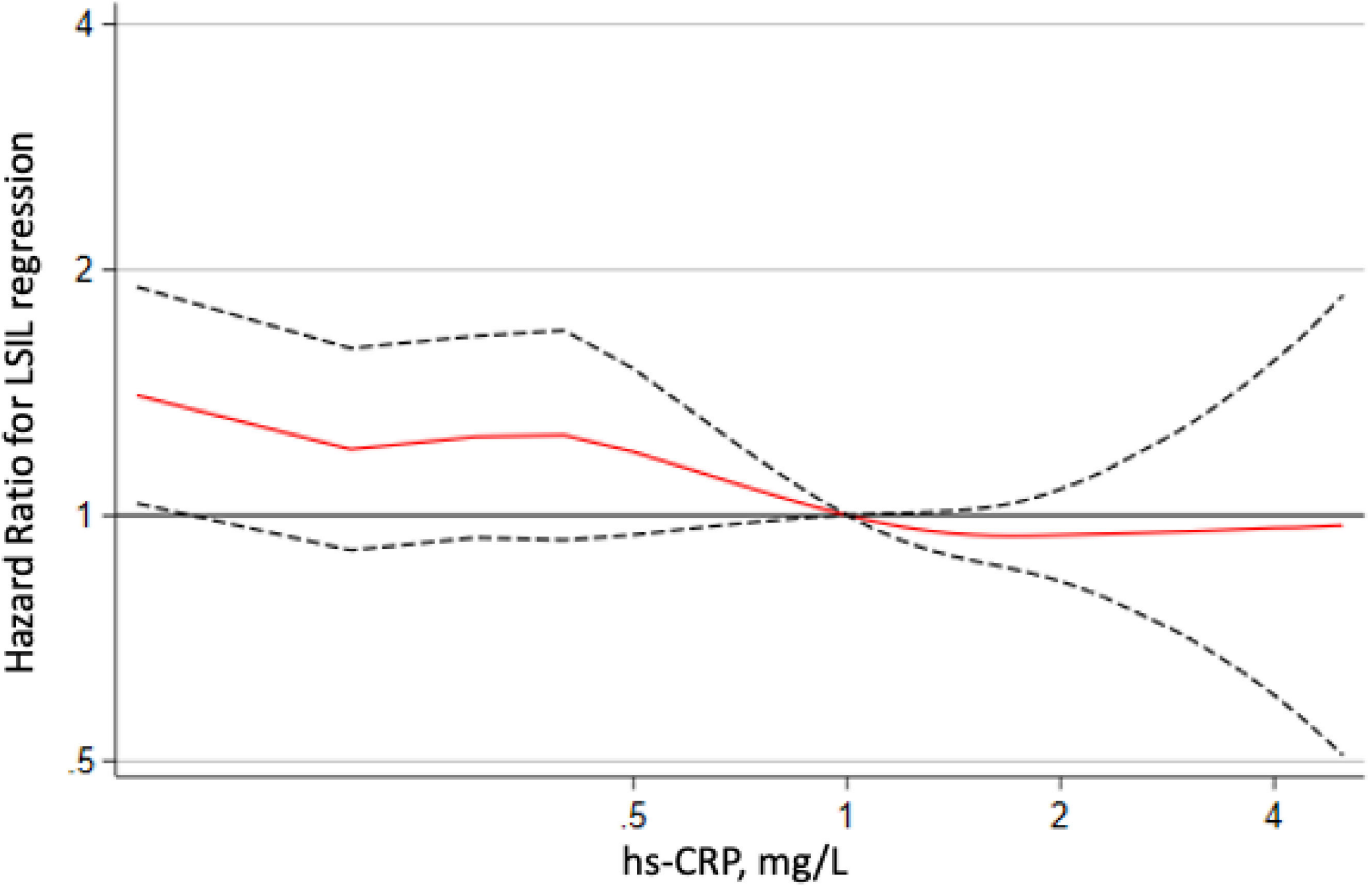
Multivariable-adjusted hazard ratios for low-grade squamous intraepithelial lesion (LSIL) regression. Curves represent adjusted hazard ratios for LSIL regression based on restricted cubic splines with knots at the 5th, 27.5th, 50th, 72.5th, and 95th percentiles of hs-CRP distribution. Models were adjusted for age, the year of a screening exam, smoking status, alcohol intake, married, HPV infection, history of diabetes, and education level. The red line represents adjusted hazard ratio, and the dashed lines represent the confidence intervals for the spline model. The horizontal black line corresponds to the normal reference hazard ratio of 1.0.

During 827.5 person-years of follow-up, 486 participants showed spontaneous regression of LSIL. A negative association between high hs-CRP level and regression was found (Table 2). After adjusting for age, the year of a screening exam, smoking status, alcohol intake, marital status, education level, HPV infection, and history of diabetes, inverse HRs for LSIL regression comparing quartiles 2–4 vs quartile 1 of hs-CRP, were 1.00 (95% CI, 0.76–1.32), 1.05 (95% CI, 0.81–1.37), and 1.33 (95% CI, 1.04–1.69), respectively (*P* for trend = 0.03). In spline regression analyses, there was a nonlinear relationship between hs-CRP levels and the adjusted HRs for LSIL regression (Figure 1). In a sensitivity analysis, we examined the association between hs-CRP level and LSIL regression after excluding 3 women with hs-CRP values greater than 10 mg/L because such elevated values are likely to be caused by acute infection or underlying medication problems (eTable 1).^45^ The results did not qualitatively change.

**Table 2.** Regression of LSIL by hs-CRP quartile

hs-CRP quartiles	Person-years	Incident cases	Incidence Density (per 100 person-years)	Multivariate HR^a^ (95% CI)
Q1 (0.1–0.2 mg/L)	385.0	231	60.0	1.00 (reference)
Q2 (0.3–0.3 mg/L)	115.2	70	60.7	1.00 (0.76–1.32)
Q3 (0.4–0.6 mg/L)	141.9	87	61.3	1.05 (0.81–1.37)
Q4 (0.7–15.1 mg/L)	185.4	98	52.9	1.33 (1.04–1.69)
*P* for trend				0.028

CI, confidence intervals; HR, hazard ratios; hs-CRP, high sensitivity C-reactive protein; LSIL, low-grade squamous intraepithelial lesion.

^a^An inverse hazard ratio (1/HR) with higher than 1.00 means “less likely to regress”. Estimated from parametric proportional hazards models adjusted for age, the year of a screening exam, smoking status, alcohol intake, married, HPV, history of diabetes, and education level.

The associations between the regression and serum hs-CRP levels were similar across participant subgroups (eTable 2). There were no significant interactions according to age (<50 vs ≥50 years), HPV infection (negative vs positive), smoking status (ever or current smoker vs never smoker), alcohol intake (<10 vs ≥10 g/day), BMI categories (<23 vs ≥23 kg/m^2^), or HOMA-IR (<2.5 vs ≥2.5).

## DISCUSSION

To our knowledge, this is the first cohort study to examine the effects of host inflammation status on spontaneous regression of incident LSIL. We found a negative association between elevated hs-CRP and LSIL regression. These outcomes suggest that low-grade inflammation could affect the natural course of LSIL, which is biologically plausible considering that inflammation has a causal role in cervical carcinogenesis.^21^^,^^22^

There were some reports on cervical precursor lesions or HPV infections associated with the perturbated host inflammatory responses.^26^^,^^28^ Prior cross-sectional analyses demonstrated that the cell-mediated immune response was decreased in the more severe cervical lesions, while overall immune activation was increased.^26^^,^^28^ Several longitudinal studies identified that the cell-mediated immune response could affect the regression of cervical lesions and/or HPV infection.^27^^,^^29^ In addition to systemic host inflammation, several studies assessed local immune responses, using inflammatory cells in the cervical specimens^31^^–^^33^ or inflammatory cytokines IL-6 and IL-8 in cervicovaginal lavages.^34^ They revealed that local immune responses increased with the severity of the cervical lesion and/or LSIL progression.^31^^–^^34^

Previous studies assessed female participants with prevalent cervical lesions, whereas our study analyzed the women with the incident LSIL, which would be a more accurate reflection of the natural history of the disease. Moreover, some of these studies used cervical specimens obtained using colposcopic biopsy,^27^^,^^31^^,^^33^^,^^34^ which is a traumatic and invasive procedure that may elicit immune responses capable of affecting the natural course of cervical precursor lesions. Besides having small sample sizes (less than 100 LSIL cases), some are cross-sectional studies,^26^^,^^28^^,^^32^^–^^34^ statistically limited by the temporal ambiguity between host immune response and the biological behavior of cervical lesions.

The exact mechanisms underlying low-grade inflammation and LSIL regression have not been fully elucidated. Persistent high-risk HPV infection is a prerequisite for the progression of high-grade lesions.^46^ Through degradation of the tumor suppressor proteins using viral oncoproteins E6 and E7, infected cervical epithelium could enter an uncontrolled cell cycle and a state of neoplastic transformation.^47^ These viral proteins are associated with the transition from chronic inflammation to cancer progression via increased activity of NF-κB.^48^^,^^49^ Subbaramaish et al examined the link between HPV oncogenes and the inflammatory cascade and demonstrated overexpression of cyclooxygenase-2 pathways in HPV-infected cell lines.^50^ Altered immune response pathways and an imbalance of inflammatory cytokine profiles have been also noted as being responsible for the clearance of HPV infection, suggesting that chronic inflammation may be an important mechanism for host immune evasion and progression of the precursor lesions.^51^^,^^52^ Interestingly, long-term immune suppression induced by human immunodeficiency virus infection is associated with a high progression rate of premalignant cervical lesions, where CD4 cell counts are a possible risk factor for LSIL progression.^53^ Taken together, altered inflammatory status and persistent HPV infection could influence the natural course of cervical precursor lesions.

Smoking, a well-established risk factor for cervical cancer, leads to perturbations in the host immune response,^54^ and smokers may develop a state of persistent low-grade inflammation. Smoking as a risk factor for cervical cancers lends support to the role of low-grade inflammation in cervical carcinogenesis. In our study, we found a negative association between elevated hs-CRP levels and LSIL regression, even after adjusting for smoking. Thus, our study provides highly reliable evidence that low-grade inflammation could affect the biological behavior of LSIL.

Based on the well-known etiology of cervical cancer, screening tests for the entire population contributed to the dramatic decline in the incidence and mortality of cervical cancer.^55^ Our findings may help to better understand asymptomatic patients with low-grade lesions and further aid in the development of secondary prevention strategies. That is, hs-CRP, which is a feasible biomarker in practice, could be used to predict at-risk groups for malignant progression in LSIL patients.

Since obesity and/or metabolic syndrome have been reported to be associated with increased risk of HPV infection or its persistence,^56^^,^^57^ we have also evaluated whether or not the association between the hs-CRP level and LSIL regression differs based on the presence of overweight or insulin resistance. The associations between the regression and serum hs-CRP levels, however, did not differ by BMI categories (<23 vs ≥23 kg/m^2^), or HOMA-IR (<2.5 vs ≥2.5). And the lack of association between hs-CRP and LSIL were observed in each subgroup, which can be explained by the small number of subgroups, which may be insufficient to establish a relationship and lead to imprecise estimates.

There are several strengths of the present study. Systemic follow-up data from a relatively large sample size allowed us to establish temporal relationships between serum hs-CRP levels and LSIL regression. Besides, we adjusted for several important potential confounders, including smoking and HPV infection, which could affect the association between hs-CRP and LSIL regression. Finally, our study population is comprised of incident LSIL women who underwent a cervical cytology test for a health screening program, thus our results may reflect the natural course of LSIL regression in a low-risk general population.

Our study has a few limitations to consider. First, a single measurement of hs-CRP may not represent an individual’s inflammatory status during persistent LSIL, since levels can be affected by diurnal cycles or stress-induced variations. Second, these results do not exclude the possibility that hs-CRP elevation is the consequence of undetected, long-latency precancerous lesions. Third, the participants in our study consisted of young and middle-aged Korean women with relatively high socioeconomic status and educational level, so the generalizability of our findings may be limited when considering other age groups or populations with a higher prevalence of comorbidities, such as common genital infections or HIV. Last, in our analysis we do not include an important variable, sexual activity, which affects the biological behavior of LSIL. That variable should depend on the subjects’ survey, so there would be a high possibility of measurement errors, such as underestimation, considering the conservative sentiment of Korean society, if sexual activity were applied in the analysis.

In conclusion, elevated hs-CRP levels show a negative association with LSIL regression, suggesting that low-grade host systemic inflammation could affect the biological behavior of LSIL. Further studies on the regression of LSIL in relation to host systemic inflammation may provide insights into the mechanisms that contribute to the development of the malignant phenotype and may suggest novel approaches to the prevention of cervical neoplasia.

## APPENDIX A. SUPPLEMENTARY DATA

eTable 1. Inverse hazard ratios^a^ (95% CIs) of LSIL regression according to hs-CRP quartile after excluding 3 women with hs-CRP value greater than 10 mg/L

eTable 2. Inverse hazard ratios^a^ (95% CIs) of LSIL regression according to hs-CRP quartile in clinically relevant subgroups
