# Supplementary material for: High-sensitivity C-reactive Protein and Regression of Low-grade Squamous Intraepithelial Lesion: The Role of Low-grade Inflammation in Cervical Carcinogenesis
Source: J Epidemiol. 2021 Dec 5;31(12):615–20. doi: 10.2188/jea.JE20200142 (PMC8593578; doi:10.2188/jea.JE20200142)
Supplement: Supplementary file 1 [file je-31-615-s001.pdf]

eTable 1. Inverse hazard ratios[a] (95% CIs) of LSIL regression according to hs-CRP quartile after excluding 3 women with hs-CRP value greater than 10 mg/L

hs-CRP quartiles	Inverse hazard ratios (95% CIs)
Q1 (0.1–0.2 mg/L)	1.00 (reference)
Q2 (0.3–0.3 mg/L)	1.00 (0.76–1.32)
Q3 (0.4–0.6 mg/L)	1.05 (0.81–1.37)
Q4 (0.7–9.9 mg/L)	1.34 (1.05–1.72)
P for trend	0.028

CI, confidence intervals; HR, hazard ratios; hs-CRP, high sensitivity C-reactive protein; LSIL, low-grade squamous intraepithelial lesion.

[a] An inverse hazard ratio (1/HR) with higher than 1.00 means "less likely to regress"

Estimated from parametric proportional hazards models. A multivariable model was adjusted for age, year of a screening exam, smoking status, alcohol intake, married, HPV, history of diabetes, and education level.

eTable 2. Inverse hazard ratios[a] (95% CIs) of LSIL regression according to hs-CRP quartile in clinically relevant subgroups

Subgroup	hs-CRP quartiles				*P* for trend	*P* for interaction
	Q1 (0.1–0.2 mg/L)	Q2 (0.3–0.3 mg/L)	Q3 (0.4–0.6 mg/L)	Q4 (0.7–15.1 mg/L)		
Age, years						0.237
<50 (n=487)	1.00 (reference)	1.04 (0.78–1.37)	1.03 (0.74–1.28)	1.27 (0.98–1.61)	0.127	
≥50 (n=33)	1.00 (reference)	0.24 (0.06–0.95)	0.74 (0.29–1.92)	0.92 (0.31–2.70)	0.440	
HPV infection						0.183
Negative (n=60)	1.00 (reference)	1.79 (0.80–4.00)	2.17 (1.02–4.55)	1.30 (0.57–2.86)	0.035	
Positive (n=417)	1.00 (reference)	0.93 (0.68–1.25)	1.02 (0.76–3.33)	1.28 (0.98–1.67)	0.124	
Smoking						0.540
Never smoker (n=416)	1.00 (reference)	1.00 (0.73–1.37)	1.09 (0.81–1.47)	1.37 (1.03–1.79)	0.038	
Ever or current smoker (n=26)	1.00 (reference)	1.05 (0.34–3.23)	2.50 (0.81–3.33)	1.96 (0.64–5.88)	<0.001	
Alcohol intake, g/day						0.345
<10 (n=402)	1.00 (reference)	1.67 (0.76–1.43)	1.14 (0.85–1.52)	1.37 (1.03–1.67)	0.025	

≥10 (n=67)	1.00 (reference)	0.88 (0.44–1.79)	0.72 (0.27–1.89)	0.69 (0.34–1.43)	0.540	
BMI, kg/m^2						0.294
<23 (n=385)	1.00 (reference)	0.88 (0.64–1.20)	0.97 (0.72–1.32)	1.43 (1.02–2.00)	0.151	
≥23 (n=134)	1.00 (reference)	1.52 (0.82–2.78)	1.35 (0.79–2.33)	1.39 (0.86–2.22)	0.341	
HOMA-IR						0.298
<2.5 (n=469)	1.00 (reference)	1.00 (0.75–1.33)	1.04 (0.79–1.37)	1.32 (1.02–1.72)	0.056	
≥2.5 (n=49)	1.00 (reference)	1.19 (0.48–2.94)	1.41 (0.62–3.23)	1.96 (0.91–4.35)	0.230	

BMI, body mass index; CI, confidence intervals; HOMA-IR, homeostasis model assessment of insulin resistance; HPV, human papillomavirus; hs-CRP, high sensitivity C-reactive protein; LSIL, low-grade squamous intraepithelial lesion.

[a] An inverse hazard ratio (1/HR) with higher than 1.00 means “less likely to regress”. Estimated from parametric proportional hazards models adjusted for age, the year of a screening exam, smoking status, alcohol intake, married, HPV infection, history of diabetes, and education level.
